# Effect of Urea Coated with Polyaspartic Acid on the Yield and Nitrogen Use Efficiency of Sorghum (*Sorghum bicolor*, (L.) Moench.)

**DOI:** 10.3390/plants11131724

**Published:** 2022-06-29

**Authors:** Peng Yan, Mengying Fang, Lin Lu, Liang Ren, Xuerui Dong, Zhiqiang Dong

**Affiliations:** Institute of Crop Sciences, Chinese Academy of Agricultural Sciences/Key Laboratory of Crop Physiology and Ecology, Ministry of Agriculture, Beijing 100081, China; yanpeng01@caas.cn (P.Y.); 82101182163@caas.cn (M.F.); lulin@caas.cn (L.L.); 20200127@mails.jlau.edu.cn (L.R.); sjtu_008@163.com (X.D.)

**Keywords:** sorghum, polyaspartic acid, grain yield, nitrogen use efficiency, North China Plain

## Abstract

Innovative approaches to enhance N fertilization to improve season-long N availability are essential to optimal sorghum (*Sorghum bicolor*, (L.) Moench.) productivity and N use efficiency. A two-year field experiment was conducted in the 2020 and 2021 summer seasons on the North China Plain to determine the effects of a novel urea coated with polyaspartic acid (PAA) (PN) and a control treatment (CN) on grain sorghum yield and N utilization characteristics at four N application rates (0, 60, 120, and 240 kg ha^−1^). The results showed that sorghum yield, agronomic traits (including leaf area duration (LAD), crop growth rate (CGR), and dry matter accumulation (DMA)), the accumulation of nitrate N and ammonium N in the 0–60 cm soil layer, stover and grain N content, and total N uptake (NUT) in 2020 and 2021 significantly increased as N application rates increased from 0 to 240 kg ha^−1^, whereas nitrogen agronomic efficiency (NAE), N uptake efficiency (NUpE), and N utilization efficiency (NUtE) varied inversely with increasing N application rates. Compared to CN, PN demonstrated a significant enhancement in grain sorghum yield, LAD, and CGR, from 3.3% to 7.1%, from 4.8% to 6.1%, and from 5.8% to 6.8%, respectively, at 60 and 120 kg N ha^−1^. PN improved the N availability (mainly nitrate-N) in the sorghum soft dough and the stover and grain N content at harvest and NUT, NUpE, and NAE accordingly compared with CN at the 60 and 120 kg ha^−1^ N application rates. In short, our two-year field trials demonstrated that PN with 120 kg N ha^−1^ is recommended in grain sorghum to optimize sorghum productivity and nitrogen use efficiency at the current yield level in the North China Plain.

## 1. Introduction

The number of undernourished people in the world affected by hunger reached 811 million in 2020, and more than 700 million undernourished people live in arid and semiarid regions of Asia and Africa [1]. Sorghum (*Sorghum bicolor*, (L.) Moench.) is quantitatively the world’s fifth largest cereal grain; moreover, it is the only viable food grain for the world’s most food insecure people [2]. It is drought-resistant and able to withstand periods of high temperature among cereal crops [3]. Hence, sorghum has the potential to end severe food insecurity, especially among countries in arid and semiarid lands.

Nitrogen (N) is one of the most important and limiting nutrient factors for crop production and contributes more than any other fertilizer to increasing crop yield and quality, including grain sorghum [4,5]. Currently, producers tend to apply excessive N to further increase crop yield, which results in massive N losses through leaching, runoff, volatilization, environmental pollution, and low N use efficiency [6,7].

Sorghum yield increased by nearly 50 kg ha^−1^ yr^−1^ in the past sixty years, and approximately 60% to 65% of the total yield gain in grain sorghum was assumed to be due to cultural practices, e.g., involving N fertilizer [4]. Previous studies have shown that an N deficit would lead to lower sorghum biomass by decreasing the leaf area duration (LAD) and the crop growth rate (CGR) [8]. The addition of N fertilizer could boost sorghum aboveground biomass accumulation and grain yield accordingly [9,10]. Typically, sorghum yield becomes less responsive to N as the yield approaches 8.5 Mg ha^−1^, and the optimal N application rate should be under 170 kg N ha^−1^ [11,12]. However, these N application rates could not improve sorghum yield alone and need to be combined with adaptive N management. Currently, in most sorghum production areas in semiarid lands of China, local farmers use basal N fertilizer combined with topdressing at approximately the sorghum five-leaf stage [13]. However, there are two main issues with this N management practice. First, it is difficult to quantify the amount of base and topdressing fertilizer due to the variation in climate conditions, soil type, and even sorghum variety. Second, topdressing timing depends largely on soil moisture conditions [14]; nevertheless, as sorghum is commonly cultivated in semiarid lands and rarely available for irrigation, topdressing practices often occur after raining and usually miss the best topdressing timing, resulting in fertilizer waste or even yield penalty.

Developing new types of urea, such as environmentally friendly nitrogen [15] and controlled-release urea (CRU) [16], and overcoming the easy volatilization and leaching of urea, are likely the most feasible ways to promote both grain yield and nitrogen use efficiency [17,18]. Recently, CRU, which was designed to prolong nutrient release duration to better match crop nutrient uptake, combined with a one-time basal application method, has been widely used in China [19,20]. However, the cost of CRU is basically higher than that of conventional urea and seems too costly for cereal production. In addition, materials used for coating urea could lead to soil pollution [21]. Therefore, developing low-cost, green, and degradable coating materials is essential for CRU extension in cereal production regions.

Polyapartate acid (PAA) is an eco-friendly and natural amino acid polymer that has been mass-produced by companies. It has shown a strong chelation, dispersion, and adsorption capacity on macro- and microelements, which has resulted in the nutrient enrichment of the soil [22]. Du et al. (2011) indicated that PAA can inhibit soil urea nitrification and ammonization, thus extending the release of urea and reducing N loss [23]. A series of studies showed that PAA can enhance N utilization and promote rice dry matter accumulation, yield, and NUE in paddy fields [24,25,26,27]. However, related knowledge regarding the effects of PAA-coated urea on sorghum yield and nitrogen utilization, especially when combined with the one-time basal application method, is still limited.

Hence, in this work, we explore the effect of PN and CN with different N application rates on sorghum yield and NUE. The specific objectives of this study were (1) to evaluate the effect of PN and CN with different N rates on mainly sorghum agronomic traits (LAD, CGR, and DMA) and (2) to investigate the effects of PN on sorghum yield and NUE, thus aiming to find new fertilizer techniques allowing for a more sustainable and highly efficient application of fertilizers in sorghum production.

## 2. Results

### 2.1. Sorghum Grain Yield

**Figure 1:**
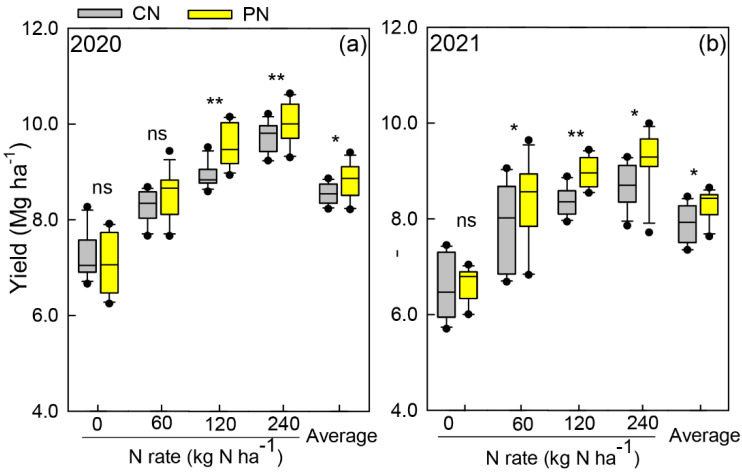
Sorghum yield at 0–240 kg N ha^−1^ nitrogen application rates under the control treatment (CN) and polyaspartic acid-coated urea (PN) in 2020 (**a**) and 2021 (**b**). * and ** represent a significant difference between CN and PN at the same N rate at the 0.05 and 0.01 levels, respectively, and ns represents no significant difference.

The year, N rates, and treatments showed significant (*p* < 0.05) effects on sorghum grain yield. As shown in Figure 1, N rates and PN significantly increased sorghum yield. In comparison with 0 kg N ha^−^^1^, the sorghum yield under 60–240 kg N ha^−^^1^ increased by 17.2–37.5% in 2020 and 22.3–34.9% in 2021. PN increased the sorghum yield by 2.8–6.9% and 6.0–7.1% in comparison with CN in 2020 and 2021, respectively, with nitrogen application rates from 60 to 240 kg N ha^−^^1^ nitrogen application rates. Moreover, among the 0–240 kg N ha^−^^1^ nitrogen application rates, PN significantly increased sorghum yield by 4.6% and 7.0% at 60 and 120 kg N ha^−^^1^, respectively, compared with CN, while it showed no significant difference with the 0 and 240 kg N ha^−^^1^ rates. These results indicated that PN promoted sorghum yield, especially under moderate N application rates.

### 2.2. Leaf Area Duration, Crop Growth Rate, and Dry Matter Accumulation

**Figure 2:**
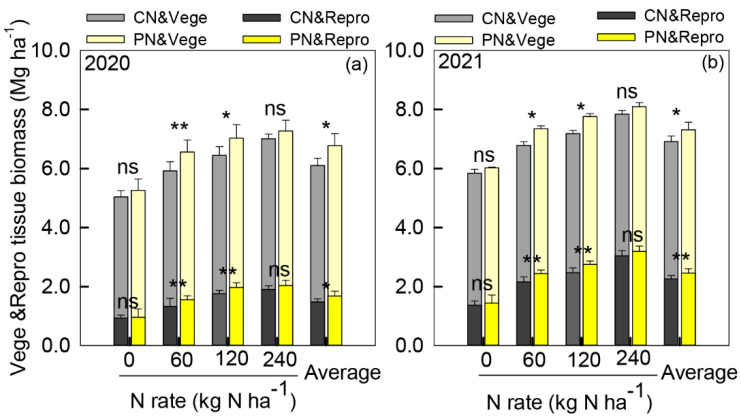
Vegetative and reproductive organ biomass at the flowering growth stage at 0–240 kg N ha^−1^ nitrogen fertilizer rates under control treatment (CN) and polyaspartic acid-coated urea (PN) in 2020 (**a**) and 2021 (**b**). * and ** represent a significant difference between CN and PN at the same N rate at the 0.05 and 0.01 levels, respectively, and ns represents no significant difference.

From the five-leaf to the flowering stage, the N application rates significantly affected the leaf area duration (LAD), the crop growth rate (CGR), and consequently the dry matter accumulation (DMA) (Table 1). Compared with 0 kg N ha^−^^1^, the LAD and CGR under 60–240 kg N ha^−^^1^ increased by 21.9–23.6% and 40.7–63.1% in 2020 and by 29.4–39.8% and 32.3–78.0% in 2021, respectively. PN increased CGR, LAD, and DMA but showed no significant difference compared with CN during this stage. Nevertheless, the reproductive tissue biomass at the flowering stage of the PN treatment under 60 and 120 kg N ha^−^^1^ was significantly (*p* < 0.05) higher than that of CN, which suggested a greater yield potential (Figure 2).

From the flowering to physiological maturity stages, N application rates and PN both showed significant (*p* < 0.05) effects on LAD, CGR, and DMA. LAD and CGR under 60–240 kg N ha^−^^1^ increased from 0.6% to 17.8% and from 18.8% to 43.9%, respectively, in 2020 and from 16.3% to 33.0% and from 30.9% to 46.1%, respectively, in 2021. In comparison with CN, PN significantly increased LAD and CGR by 6.1% and 6.8% with 60 kg ha^−^^1^ and 4.8% and 5.8% with 120 kg ha^−^^1^, respectively, during the two experimental years (Table 1).

### 2.3. Topsoil Mineral Nitrogen Content

**Figure 3:**
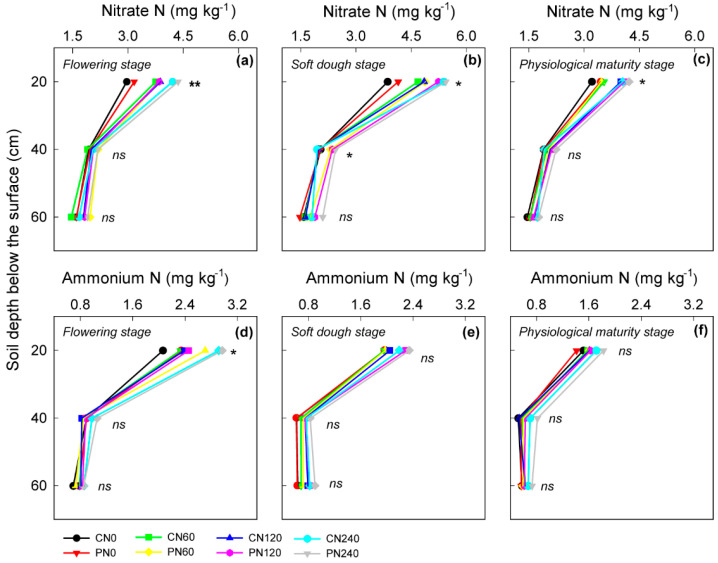
Effects of the control treatment (CK) and polyaspartic acid-coated urea (PN) on 0–60 cm topsoil nitrate N (**a**–**c**) and ammonium N (**d**–**f**) during the sorghum flowering (**a**,**d**), soft-dough (**b**,**e**), and physiological maturity (**c**,**f**) stages at 0−240 kg N ha^−1^ nitrogen application rates in 2020 and 2021. * and ** represent a significant difference between CN and PN at the 0.05 and 0.01 levels, respectively, and ns represents no significant difference.

As shown in Figure 3, from flowering to physiological maturity, the soil mineral nitrogen content (including nitrate N and ammonium N) significantly decreased over time. N application rates and PN both showed significant (*p* < 0.05) effects on soil nitrate-N content and total mineral nitrogen content (nitrate N plus ammonium N), whereas no significant difference was detected in ammonium N among treatments. The topsoil nitrate-N content under the 60–240 kg N ha^−^^1^ treatment increased from 6.8% to 16.2%, from 1.4% to 5.3%, and from 4.4% to 7.7% in the 0–20 cm, 20–40 cm and 40–60 cm soil layers, respectively, compared with 0 kg N ha^−^^1^. PN significantly increased the 20–40 cm and 40–60 cm soil NO_3_^−^-N contents at the flowering and soft dough stages compared with CN at the rates of 60 and 120 kg N ha^−^^1^.

### 2.4. N Content

**Figure 4:**
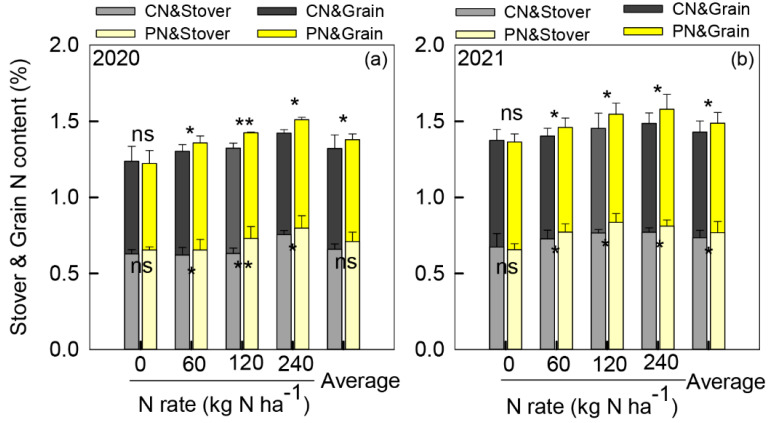
Sorghum stover and grain N content at the physiological maturity stage at 0–240 kg ha^−1^ nitrogen rates under control treatment (CN) and polyaspartic acid-coated urea (PN) in 2020 (**a**) and 2021 (**b**). * and ** represent significant differences between CN and PN at the same N rate at the 0.05 and 0.01 levels, respectively, and ns represents no significant difference.

As shown in Figure 4, the stover and grain N contents at harvest significantly (*p* < 0.05) increased with increasing N rates. In comparison with 0 kg ha^−^^1^, stove and grain N contents under 60–240 kg N ha^−^^1^ increased from 0 to 21.0% and from 8.1% to 19.2% in 2020 and from 12.8% to 20.8% and from 4.5% to 11.9% in 2021, respectively. Compared with the CN stove, the N contents of 60, 120, and 240 kg N ha^−^^1^ with PN increased by 5.7%, 12.0%, and 5.5%, respectively. The grain N content of the varying N applications with PN increased by 4.1%, 7.0%, and 6.3%, respectively.

### 2.5. Nitrogen Use Efficiency

At harvest, as shown in Table 2, N application rates and PN had significant effects on sorghum NUE. N application rates increased NUT but sharply decreased AE, NUpE, and NUtE. In comparison with 0 kg ha^−^^1^, NUT with 0–240 kg ha^−^^1^ increased by 29.2–68.1% in 2020 and 29.5–70.0% in 2021. Compared with CN, PN increased NUT, NAE, and NUpE by 7.4%, 15.7%, and 22.8% in 2020 and 10.4%, 29.9%, and 39.9% in 2021, respectively; however, it showed no significant effect on NUtE.

## 3. Discussion

In the current study, the sorghum grain yield of PN was significantly higher than that of CN at the same N application rate. Several previous studies on other cereal crops showed similar trends, which indicated that PN significantly increased maize and rice yields under field conditions [25,27,28]. In this study, sorghum yield under PN increased by 4.3% in 2020 and by 6.5% in 2021 compared with that under CN. This may be due to the cloudy and rainy weather conditions during the sorghum flowering stage in 2021, which could partly explain the yield gap between the two experimental years. Overall, sorghum yield increased as nitrogen input increased from 0 to 240 kg N ha^−1^; moreover, PN showed a significant advantage in sorghum yield with 60 and 120 kg N ha^−1^, while there was no significant difference as the N rate increased to 240 kg N ha^−1^. Li et al. (2018) suggested that the sorghum yield in the North China Plain averaged 8.3 Mg ha^−1^ [29], and the recommended nitrogen application rate ranged from 75 to 150 kg ha^−1^ [30]. These results indicated that PN with 120 kg N ha^−1^ had the greatest effect on sorghum grain yield. This was consistent with the findings of Schlegel and Havlin (2021), who reported that the N rate for the maximum grain sorghum yield would have been 110 kg N ha^−1^ based on 55 years of continuous field observation [31].

Basically, increasing nitrogen inputs can accordingly extend the leaf area duration (LAD), promote the crop growth rate (CGR), and increase dry matter accumulation (DMA) accordingly [8,32]. In the present study, LAD, CGR, and DMA increased with N addition, while PN showed no significant effect on these traits from the five-leaf stage to the flowering stage. However, from flowering to the physiological maturity stage, PN increased LAD, CGR, and DMA at rates of 60 and 120 kg N ha^−^^1^ compared with CN. This may be partly due to PN delaying the release rate of urea. On the one hand, PAA is a good biodegradable chelant, especially for soil copper (Cu) and zinc (Zn) [33], and Lu et al. (2016) indicated that PAA could retard the nitrogen release rate effectively [34], thus decreasing the apparent N supply and reducing DMA compared with CN during the vegetative growth stage. On the other hand, the slow release of PN increased the post-flowering soil-apparent N supply and promoted LAD, CGR, and DMA accordingly.

Nitrate nitrogen and ammonia nitrogen are the two main forms of soil inorganic N. Deng et al. (2015) showed that PN had a positive effect on rice N utilization and the soil N balance in paddy fields [27]; moreover, PAA reduced the apparent N supply before flowering and enhanced the apparent N supply post-flowering, which led to a better synchronization between the N supply and the plant N demand. In this study, compared with CN, the PN treatment significantly increased the content of nitrate N, ammonia N, and mineral N in the 0–20 cm and 20–40 cm layers of topsoil at the flowering and soft dough stages but showed no significant difference at physiological maturity (Figure 3). This finding is consistent with Du et al. (2011), who reported that PAA could reduce nutrient loss while increasing the enrichment of soil nutrients [23]. A high N input could significantly increase the soil inorganic N content, especially in the topsoil layer [35]. As mentioned earlier, the N rate for the maximum grain sorghum yield would have been no more than 110 kg N ha^−^^1^ for maximum grain sorghum yield [31], and the extensive use of N fertilizer at 240 kg ha^−^^1^ markedly increased soil inorganic N (including nitrate and ammonia nitrogen) accumulation within the 0−60 cm soil layer in the present study. According to Adam et al. (2015), sorghum was capable of extracting relatively high quantities of soil N under N-limiting conditions [36]; in this case, an N application rate under 120 kg ha^−1^, especially for PN, would satisfy the grain sorghum N demand at the current yield level and reduce soil nutrient losses. Deng et al. (2014) indicated that PN could improve soil mineral N content by controlling the release of nitrogen and reducing the leaching of nitrate nitrogen and ammonia nitrogen [26]; Liu et al. (2019) further proved that PAA could promote root development and nitrate absorption [37]. Therefore, the increase in the accumulation of soil inorganic nitrogen could mainly be attributed to both the controlled release of urea before pre-flowering and the high nitrogen utilization rate post-flowering.

Typically, plants with controlled released fertilizer can increase nitrogen absorption and utilization, which together increase N use efficiency (NUE) [38,39,40]. In the current study, PN significantly increased the stover and grain N content, NUT, NAE, and NUpE at 60 and 120 kg N ha^−^^1^. There are currently two main explanations concerning this. First, PAA is a hydrophilic and biodegradable polymer with a good dispersibility and adsorption capacity [41], and several previous studies have pointed out its capability in retarding the nitrogen release rate and promoting N utilization [24,25,26,27,34]. Second, Wang et al. (2018) proved that PAA can increase maize nitrogen assimilation by enhancing nitrate reductase activity under hydroponic conditions [42]. However, PAA showed no significant effect on N utilization efficiency (NUtE) in this study. These results revealed that PAA-promoted sorghum NUE was mainly attributed to the controlled release of urea, which enhances the N supply to meet plant N demand during the grain-filling stage.

## 4. Materials and Methods

### 4.1. Field Experimental Design

Field experiments were performed in the 2020 and 2021 summer seasons in the North China Plain at Xinxiang Experimental Station (35°16′ N, 113°80′ E) of the Institute of Crop Science, Chinese Academic of Agricultural Science. The soil type of the experimental field is a clay loam, and the cropping system is a winter wheat–sorghum rotation system. The initial status of the 0–60 cm topsoil at the experimental site is given in Table 3.

**Figure 5:**
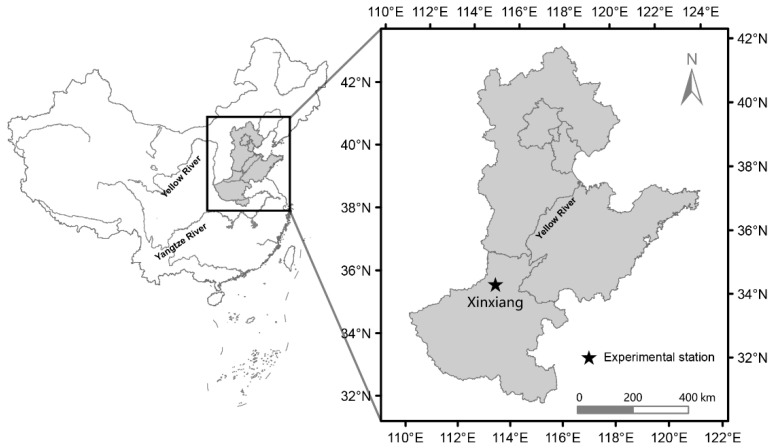
Location of Xinxiang Experimental Station and the North China Plain.

The daily average air temperature from planting to harvest was 24.8 °C in 2020 and 24.5 °C in 2021, and the cumulative rainfall during the whole sorghum growing season was 455.0 mm in 2020 and 1306.5 mm in 2021. Generally, the climate in the 2020 sorghum growing season was warmer and drier than that in 2021 (Figure 5).

Experimental plots were distributed in a randomized complete block design with four replicates. The treatments included two urea types, a control treatment (CN, uncoated urea), and urea coated with PAA (PN), and both urea types included four N application rates: 0, 60, 120, and 240 kg N ha^−^^1^. PN was made in the laboratory in two steps. First, approximately 530 g of PSI was mixed with approximately 1000 mL of distilled water, and NaOH was added until PSI was completely dissolved; thus, a PAA solution was obtained. Second, urea was coated with the PAA solution at approximately 0.3% of the total urea rate with a mixer, and the mixture was air-dried naturally in the shade.

The plot size was 56 m^2^ (7 m wide and 8 m long). Ca(H_2_PO_4_)_2_·H_2_O and K_2_SO_4_ were broadcast for each plot prior to planting, providing sources of 75 kg P ha^−1^ and 35 kg K ha^−1^, respectively. Irrigation water was applied once after planting. Herbicides (premix of atrazine and metolachlor, Jintian Tech Co., Ltd., Liaoning, China) were sprayed after sowing but prior to emergence. Weeds in plots were removed by hand at the stem elongation stage. Pests and diseases were well controlled by spraying insecticide (Beta-cyfluthrin, Shanghai Hulian Biological Pharmaceutical Co., Ltd., Xiayi, China) and fungicide (Cabrio, BASF (China) Co., Ltd., Shanghai, China) at the stem elongation and anthesis stages. No obvious weed, pest, or disease stress was observed during the two experimental seasons.

### 4.2. Meteorological Data and Growth Stages

Meteorological data, including daily air temperature and daily cumulative precipitation, were obtained from the local meteorological station. Sorghum growth stages were recorded when more than 50% of the sorghum in the plot was within a specific period according to Vanderlip and Reevesthe [43]. The phenological phases of sorghum are shown in Table 4.

### 4.3. Agronomic Traits

Twenty consecutive plants in the central two rows of each plot were tagged for measurement at the 5-leaf, flowering, and physiological maturity stages. At each growth stage, four sorghum plants in each plot were cut at ground level, the length and width of each green leaf were measured, and the leaf area was calculated by adding the individual leaf area (length × width × 0.75) and then multiplying by the plant density (plants m^−2^). After that, the plant tissues of each plot were dried at 65 °C to a constant weight and measured with a 0.01 g precision balance. The aboveground dry matter accumulation (DMA) was calculated as the plant biomass multiplied by the plant population [44]. Leaf area duration (LAD) and crop growth rate (CGR) were calculated by the following formulas:

LAD = [(LA_2_ + LA_1_) × (T_2_ − T_1_)]/2 (1)

where LA and T are leaf area and sample date, respectively;

CGR = (W_2_ − W_1_)/(T_2_ − T_1_) (2)

where W, LA, and T are aboveground biomass, leaf area, and sample date, respectively.

### 4.4. Sorghum Grain Yield

At harvest, 12 m^2^ in the inner four rows of each plot was harvested to measure grain yield. Plant density was measured as the number of plants per square meter. Grain moisture content was determined using a PM-8188 grain moisture analyzer (Tokyo, Japan) and measured 10 times for each sample. Grain from the sampling area was weighed and corrected to a water content of 15.5% for the final sorghum grain yield.

### 4.5. Soil Mineral Nitrogen Content

At the flowering, soft dough, and physiological maturity stages, composite soil samples were randomly collected from five cores per plot to a depth of 60 cm and in 20 cm increments. Fresh soil samples of each plot from the same soil depth were mixed by hand as a composite sample and temporarily stored in a refrigerator at 4 °C prior to chemical analysis. Field-moist soil samples were weighed and extracted with a 2 M KCl solution and filtered, and the aliquots were analyzed for concentrations of nitrate N and ammonium N using an AA3 Autoanalyzer (Hamburg, Germany).

### 4.6. Stover and Grain N Content

Dry samples of stover and grain at the physiological maturity stage were collected and ground to determine the stover tissue and sorghum kernel N concentrations using an Elementar Analyzer (Elementar vario Pyro Cube, Langenselbold, Germany) [45]. The stover and grain N contents were calculated as the product of the N concentration and the dry weight of each part. Total N uptake (NUT), N agronomic efficiency (NAE), N uptake efficiency (NUpE), and N utilization efficiency (NUtE) were calculated by the following formulas:

NUT (kg ha^−1^) = GY × GrainN + Stover × StoverN (3)

NAE (kg kg^−1^) = (GYn − GY_0_)/N (4)

NUpE (%) = (NUT_n_ − NUT_0_)/N (5)

NUtE (kg ka^−1^) = (GY_n_ − GY_0_)/(NUT_n_ − NUT_0_) (6)

where GrainN and StoverN are the kernel and stover (without the kernel) N content at harvest, GY_N_ and GY_0_ are the grain yield at maturity with and without N input, respectively, N is the nitrogen input, and NUT_N_ and NUT_0_ are the total N uptake at maturity with and without N input, respectively [46].

### 4.7. Statistical Analyses

Analysis of variance was performed with IBM SPSS Statistics 25.0 (IBM Inc., Armonk, NY, USA) to test the effects of year, treatments (CN and PN), and N rates on sorghum yield, agronomic traits, topsoil mineral nitrogen content, N content, and nitrogen use efficiency. Mean values were tested using the least significant differences (LSD) test at the 5% (*p* < 0.05) probability level. Figures were prepared using Sigmaplot 12.5 and SigmaStat 4.0 (Systat Software, Inc., San Jose, CA, USA).

## 5. Conclusions

In the present study, we investigated the effect of a novel fertilizer synergist, PAA, on sorghum yield and nitrogen use efficiency. It was concluded that (1) PN increased sorghum yields at rates of 60 and 120 kg N ha^−^^1^ by promoting CGR, LAD, and DMA from the flowering stage to the physiological maturity stage compared to CN; (2) PN increased the soil inorganic N content and provided an improved N supply compared to CN at 60 and 120 kg ha^−^^1^ N application rates; and (3) PN increased the total N uptake (NUT) and N agronomic efficiency (NAE), mainly due to the increased N uptake efficiency (NUpE) relative to CN. Therefore, urea coated with PAA at an application rate of 120 kg ha^−^^1^ N would be suitable for sorghum to improve sorghum yield and nitrogen use efficiency.

## Figures and Tables

**Table 1:** Sorghum agronomic traits during the 5-leaf-flowering and flowering-physiological maturity growing stages at four nitrogen fertilizer rates under control treatment (CN) and polyaspartic acid-coated urea (PN) in 2020 and 2021.

Year	N Rate	Treaments	Growth Stages					
			5-Leaf–Flowering			Flowering–Physiological Maturity		
			LAD ^‡^	CGR	DMA	LAD	CGR	DMA
	kg ha^−1^		m^2^ d^−1^	kg ha^−1^ d^−1^	kg ha^−1^	m^2^ d^−1^	kg ha^−1^ d^−1^	kg ha^−1^
2020	0	CN	53.8 b ^†^	10.6 b	2750.9 c	178.5 c	5.8 d	5545.1 d
		PN	50.9 b	10.3 b	2666.0 c	184.2 b	6.1 d	5850.6 d
	60	CN	65.8 a	15.2 a	3948.2 b	177.1 c	6.8 c	6483.3 cd
		PN	63.1 a	14.1 a	3674.3 b	187.9 b	7.3 b	7052.7 c
	120	CN	63.0 a	16.5 a	4281.0 a	199.6 b	7.5 b	7182.7 c
		PN	64.6 a	15.9 a	4145.8 a	214.3 a	7.9 ab	7622.1 b
	240	CN	64.8 a	17.4 a	4524.5 a	208.2 a	8.5 a	8160.6 a
		PN	64.6 a	16.6 a	4309.2 a	219.3 a	8.6 a	8234.6 a
2021	0	CN	54.1 b	7.8 c	3127.4 d	149.2 d	5.4 c	5103.6 d
		PN	52.0 b	9.3 c	3724.7 d	156.8 d	5.4 c	5054.8 d
	60	CN	69.1 a	11.5 b	4587.2 c	172.7 c	6.9 bc	6491.0 c
		PN	68.3 a	11.2 b	4477.2 c	183.2 b	7.2 b	6808.3 b
	120	CN	73.9 a	14.2 ab	5694.9 b	194.5 b	7.4 ab	6946.8 b
		PN	68.4 a	13.4 b	5346.4 b	198.5 a	7.8 a	7326.4 a
	240	CN	74.3 a	15.3 a	6136.9 a	200.6 a	7.8 a	7288.1 a
		PN	74.0 a	15.1 a	6058.1 a	206.3 a	8.0 a	7555.7 a
ANOVA								
Year			**	*	***	*	*	**
N rate			*	***	***	***	***	***
Treatments			Ns *^§^*	ns	ns	*	**	**

^†^ The LSD at *p* < 0.05 is used to compare the treatment means within the same year; means within the same year followed by the same letter are not significantly different. ^§^ ns represents no significant difference; *, **, and *** represent significant differences at the 0.05, 0.01, and 0.001 levels. ^‡^ LAD, CGR, and DMA represent leaf area duration, crop growth rate, and dry matter accumulation, respectively.

**Table 2:** Sorghum total nitrogen uptake (NUT), N agronomic efficiency (NAE), N utilization efficiency (NUtE) and N uptake efficiency (NUpE) at 0–240 kg ha^−^^1^ nitrogen fertilizer rates under control treatment (CN) and polyaspartic acid-coated urea (PN) in 2020 and 2021.

Year	N Rate	Treatments	NUT ^‡^	NAE	NUtE	NUpE
	kg ha^−1^		kg ha^−1^	kg kg^−1^	kg kg^−1^	%
2020	0	CN	115.9 d ^†^			
		PN	116.9 d			
	60	CN	147.5 c	34.3 a	66.8 a	52.6 b
		PN	153.2 c	38.3 a	63.2 a	60.7 a
	120	CN	163.9 b	22.7 b	57.3 ab	40.0 c
		PN	182.4 a	28.5 a	53.9 b	54.6 a
	240	CN	189.1 a	14.7 c	53.1 b	30.5 d
		PN	202.1 a	16.2 c	49.4 c	35.5 d
2021	0	CN	114.0 d			
		PN	110.9 d			
	60	CN	140.1 c	21.1 a	50.2 a	43.4 b
		PN	151.2 c	28.4 a	46.8 a	67.1 a
	120	CN	167.9 b	14.7 b	32.8 b	44.9 b
		PN	189.0 a	19.2 ab	32.3 b	59.3 a
	240	CN	181.2 a	8.6 c	30.3 b	28.0 d
		PN	200.4 a	10.7 c	28.7 b	37.3 c
ANOVA						
Year			ns *^§^*	*	***	ns
N rate			***	***	**	***
Treatments			**	***	ns	**

^†^ The LSD at *p* ≤ 0.05 is used to compare the treatment means within the same year; means within the same year followed by the same letter are not significantly different. ^§^ ns represents no significant difference; *, ** and *** represent significant differences at the 0.05, 0.01, and 0.001 levels. ^‡^ NUT, NAE, NutE, and NUtE represent total N uptake, nitrogen agronomic efficiency, N uptake efficiency, and N utilization efficiency, respectively.

**Table 3:** The 0−60 cm topsoil characteristics of sorghum plots prior to planting in 2020 and 2021.

Year	Soil Layers	pH	Bulk Density	Organic Matter	Total N	Nitrate N	Ammonium N	Available P	Available K
	cm		g cm^−3^	g kg^−1^	g kg^−1^	mg kg^−1^	mg kg^−1^	mg kg^−1^	mg kg^−1^
2020	0–20	7.9	1.3	12.6	1.20	4.2	1.6	15.9	114.2
	20–40	8.1	1.58	10.8	0.85	2.7	0.71	14.3	107.2
	40–60	8.1	1.64	4.2	0.42	1.6	0.62	12.5	99.1
2021	0–20	7.8	1.29	11.8	1.10	4.1	1.8	15.6	112.3
	20–40	8.1	1.56	9.8	0.67	2.5	0.8	14.1	96.5
	40–60	8.1	1.64	4.6	0.51	1.8	0.6	11.8	87.8

**Table 4:** Phenological phases of sorghum (*Sorghum bicolor*, (L.) Moench.) based on Vanderlip and Reeves (1972) [43] in 2020 and 2021.

Growth Stages	Experimental Year (Days after Sowing, d)	
	2020	2021
Sowing	23-Jun (0)	24-Jun (0)
Seedling	30-Jun (7)	28-Jun (5)
Five-Leaf	25-Jul (32)	23-Jul (29)
Flowering	22-Aug (60)	24-Aug (61)
Soft Dough	15-Sep (84)	23-Sep (91)
Physiological Maturity	10-Oct (109)	15-Oct (113)

## Data Availability

All data included in the main text.
